# RAfilter: an algorithm for detecting and filtering false-positive alignments in repetitive genomic regions

**DOI:** 10.1093/hr/uhac288

**Published:** 2022-12-29

**Authors:** Jinbao Yang, Xianjia Zhao, Heling Jiang, Yingxue Yang, Yuze Hou, Weihua Pan

**Affiliations:** College of Informatics, Huazhong Agricultural University, Wuhan 430070, China; Shenzhen Branch, Guangdong Laboratory of Lingnan Modern Agriculture, Genome Analysis Laboratory of the Ministry of Agriculture and Rural Affairs, Agricultural Genomics Institute at Shenzhen, Chinese Academy of Agricultural Sciences, Shenzhen 518120, China; Shenzhen Branch, Guangdong Laboratory of Lingnan Modern Agriculture, Genome Analysis Laboratory of the Ministry of Agriculture and Rural Affairs, Agricultural Genomics Institute at Shenzhen, Chinese Academy of Agricultural Sciences, Shenzhen 518120, China; Zhengzhou Research Base, State Key Laboratory of Cotton Biology, School of Agricultural Sciences, Zhengzhou University, Henan Zhengzhou, 450001, China; Shenzhen Branch, Guangdong Laboratory of Lingnan Modern Agriculture, Genome Analysis Laboratory of the Ministry of Agriculture and Rural Affairs, Agricultural Genomics Institute at Shenzhen, Chinese Academy of Agricultural Sciences, Shenzhen 518120, China; Shenzhen Branch, Guangdong Laboratory of Lingnan Modern Agriculture, Genome Analysis Laboratory of the Ministry of Agriculture and Rural Affairs, Agricultural Genomics Institute at Shenzhen, Chinese Academy of Agricultural Sciences, Shenzhen 518120, China; Shenzhen Branch, Guangdong Laboratory of Lingnan Modern Agriculture, Genome Analysis Laboratory of the Ministry of Agriculture and Rural Affairs, Agricultural Genomics Institute at Shenzhen, Chinese Academy of Agricultural Sciences, Shenzhen 518120, China; College of Informatics, Huazhong Agricultural University, Wuhan 430070, China; Shenzhen Branch, Guangdong Laboratory of Lingnan Modern Agriculture, Genome Analysis Laboratory of the Ministry of Agriculture and Rural Affairs, Agricultural Genomics Institute at Shenzhen, Chinese Academy of Agricultural Sciences, Shenzhen 518120, China

## Abstract

Telomere to telomere (T2T) assembly relies on the correctness of sequence alignments. However, the existing aligners tend to generate a high proportion of false-positive alignments in repetitive genomic regions which impedes the generation of T2T-level reference genomes for more important species. In this paper, we present an automatic algorithm called RAfilter for removing the false-positives in the outputs of existing aligners. RAfilter takes advantage of rare *k-*mers representing the copy-specific features to differentiate false-positive alignments from the correct ones. Considering the huge numbers of rare *k-*mers in large eukaryotic genomes, a series of high-performance computing techniques such as multi-threading and bit operation are used to improve the time and space efficiencies. The experimental results on tandem repeats and interspersed repeats show that RAfilter was able to filter 60%–90% false-positive HiFi alignments with almost no correct ones removed, while the sensitivities and precisions on ONT datasets were about 80% and 50% respectively.

## Introduction

*De novo* genome assembly is one of the fundamental problems in genomics. For a long time, the assembly of large eukaryotic genomes is challenging due to the high complexity of repetitive regions such as centromeres, telomeres, ribosomal DNA and transposons [1]. The missing of these repetitive genomic regions leads to incomplete reference genomes, which not only impede the functional studies of the repeat-related genes and cause data analytic mistakes like false-positive variant calls. Due to the advantages in accuracy and length, the generation of PacBio high-fidelity (HiFi) long sequences [2] and Oxford Nanopore Technology (ONT) ultra-long (UL) sequences provide an opportunity for solving the problem of repeat assembly and producing totally complete (also called T2T, short for telomere to telomere) reference genomes. So far, a few new assemblers have been developed to take advantage of one or both of them to generate higher-quality genomes [3–6], and a list of T2T or near-T2T reference genomes have been built in recent years with these new data and tools [7–10].

Although these new tools have significantly improved the correctness and completeness of assemblies compared with the short read- or traditional long read- based assemblers, the generation of T2T-level chromosomes still needs a lot of manual or automatic works of gap filling in repetitive regions which may cause many mis-assemblies due to the false-positive alignments between repeat copies with high similarity. Theoretically, to detect false-positive alignments, one needs to decide if the aligned sequences are from the same genomic region or different regions with similar copies by evaluating the matching of copy-specific features such as single nucleotide polymorphisms (SNPs) and structural variations (SVs). Due to the difficulty and high computational cost of SNP and SV identification and matching evaluation, the existing alignment tools are not able to take fully advantage of copy-specific features to remove false-positive alignments, so that a large part of which still exist in their outputs.

In this paper, we present a novel algorithm called RAfilter (short for repeat alignment filter) for further removing the false-positive alignments left in the outputs of existing alignment tools. Instead of removing the alignments in hard way, RAfilter assigns a kMAPQ (similar with MAPQ used in minimap2 [11]) score for each alignment which represents the possibility of false-positive and the users is able to set an appropriate threshold to remove the alignments with kMAPQ below it. To achieve this goal, RAfilter uses rare *k-*mer (subsequence of length *k* which appear in whole genome for small number of times) to represent copy-specific feature so that different types of variants can be processed in a unified way. Then the kMAPQ can be calculated by evaluating the similarity of rare *k-*mer profiles of aligned sequences including the order and sequence of rare *k-*mers. Considering the huge numbers of rare *k-*mers in large eukaryotic genomes, a series of high-performance computing (HPC) techniques such as multi-threading and bit operation are used in RAfilter to improve the time and space efficiencies. We tested the performance of RAfilter in tandem repeats and interspersed repeats on both simulated and real datasets, and the experimental results show that it was able to effectively detect the false-positive alignments.

**Figure 1:**
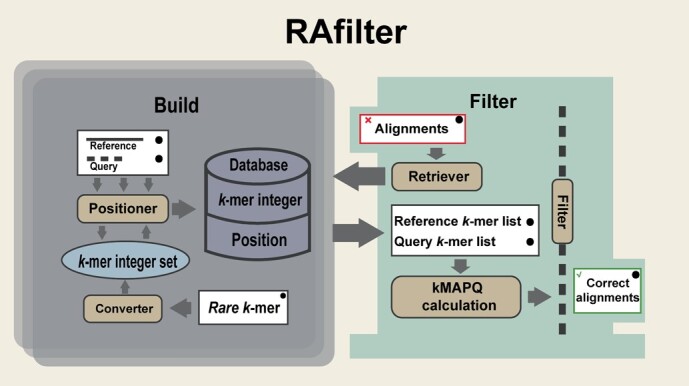
The schematic diagram of RAfilter. The left “Build” panel shows the module that builds the database of *k*-mer positions; the right “Filter” panel shows the module that uses the database of *k*-mer positions to carry out alignment filtering; the whilt rectangles and the brown rounded rectangles represent the data and operations respectively.

## Experimental results

We tested the accuracy and efficiency of RAfilter on simulated and real datasets with both PacBio HiFi and ONT reads. The simulated datasets of only repetitive regions were generated from the human T2T reference genome (chm13 v2.0) which is the existing most high-quality reference genome containing the complete and accurate tandem and interspersed repeats in human genome. The rice genome of J4155S was used to simulate the whole genome sequencing datasets and generate a real dataset.

### Accuracy on tandem repeats

The human T2T reference genome (chm13 v2.0 [7]) was downloaded from National Center for Biotechnology Information (NCBI) GenBank accession numbers GCA_009914755.4. We obtained the sequences in centromere regions in chromosome 1 and 2 from the reference genome according to the corresponding coordinates shown in their paper respectively, and then generated the reads separately using a widely-used long-read simulator PBSIM [13] and its newer version PBSIM2 [14]. The HiFi reads were generated by PBSIM with command “pbsim -data-type CCS --depth 30 --sample-fastq”, and the ONT reads were by PBSIM2 with command “pbsim2 -depth 30 --sample-fastq”. We aligned the HiFi or ONT reads to the corresponding centromere sequences by minimap2. which is the most widely-used long read aligner with parameters “-t 32 -map-pb/map-ont -o”, and usedRAfilter to filter the false-positive alignments in the output of minimap2. To build the “ground truth”, we compared the alignment starting coordinates on reference genome with the true coordinates provided by read simulators. The HiFi alignments with the differences between these two coordinates greater than 10 and the ONT alignments with the differences greater than 30 were seen as the false-positive alignments.

**Figure 2:**
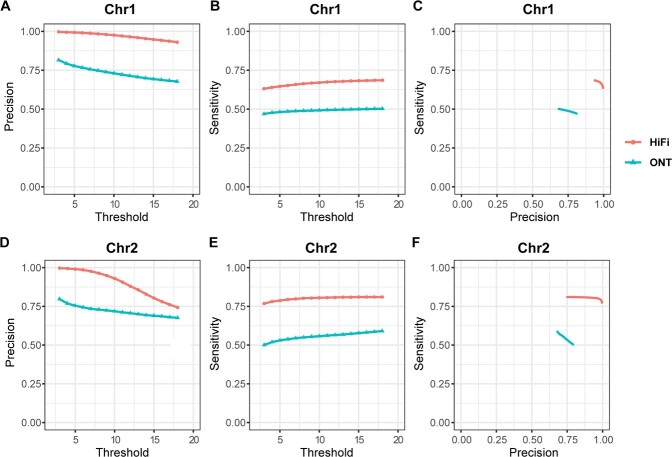
Accuracy of RAfilter on tandem repeats. The “threshold” in the figure represents a threshold, the alignments with kMAPQ below which were filtered. **A**, **B** and **C** are results on chromosome 1, while **D**, **E** and **F** are those on chromosome 2.

Fig. 2 shows that, with HiFi alignments, the precisions achieved almost 100% while the sensitivities were more than 60% and 80% on chromosome 1 and 2 respectively, which means RAfilter was able to detect most of the false-positive alignments without wrong detections. Although the results on ONT data were worse than HiFi, the sensitivities and precisions achieved about 50% and 80% respectively.

**Figure 3:**
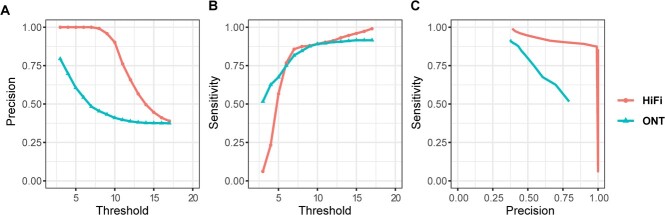
Accuracy of RAfilter on interspersed repeats. The “threshold” in the figure represents a threshold, the alignments with kMAPQ below which were filtered.

### Accuracy on interspersed repeats

To generate the simulated dataset on interspersed repeats, we randomly obtained a sequence of 100 kb from chromosome 17 of the human T2T reference genome (chm13 v2.0), and inserted 10 copies of it into chromosome 18 of the same reference genome at random positions as the interspersed repeats. These positions were recorded for assessment. To generate the copies, the substitutions and indels were added into the original sequence in the following way. First, we randomly selected 3% nucleotides and randomly changed each of them to one of the other three nucleotides with uniform probabilistic distribution. In the sequence with substitutions added, 1% nucleotides were randomly selected and removed. The HiFi and ONT reads were simulated for the whole modified chromosome 18 by the same tools with the same parameters as the experiments on tandem repeats, and were aligned by minimap2 with parameters “-t 32 -map-pb/map-ont -o”. Also, the “ground truth” was generated in the same way as tandem repeats. RAfilter was run on the alignments in the interspersed repeats (10 copies).

Fig. 3 shows similar results on interspersed repeats as tandem repeats. With HiFi alignments, RAfilter was able to filter about 90% false-positive alignments without removing correct ones. With ONT alignments, the precision and sensitivity achieved about 80% and 50% respectively.

**Figure 4:**
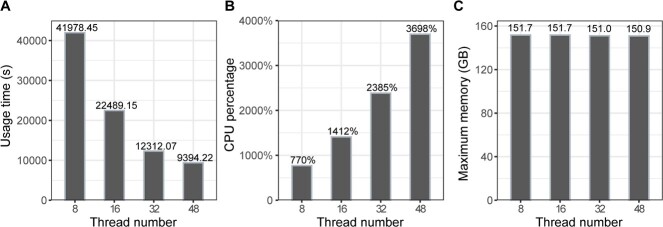
Time and space efficiency of RAfilter. **A**. Running times of RAfilter with different numbers of thread. **B**. CPU usage percentages of RAfilter with different numbers of thread. ***C**. Maximum* memory usage of RAfilter with different numbers of threads.

**Figure 5:**
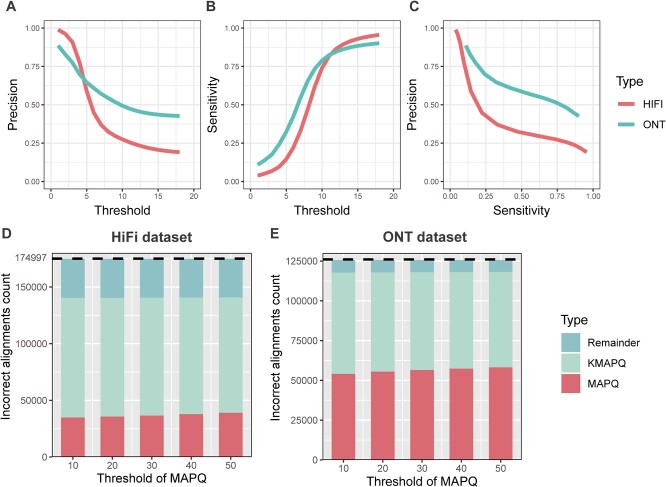
Evaluation on the whole genome. **A, B, C.** Accuracy of RAfilter on whole genome datasets of rice; the “threshold” represents a threshold, the alignments with kMAPQ below which were filtered; **D, F.** Performance of RAfilter on the alignments left from the MAPQ based filtering; red shows the proportion of the false-positive alignments removed by MAPQ based filtering; shallow blue shows the proportion that was not removed by MAPQ based filtering, but can be removed by RAfilter; dark blue shows the proportion left.

### Time and space efficiency

To test the time and space efficiencies of RAfilter, we downloaded the whole-genome real ONT reads “rel6” of the human chm13 cell line from https://github.com/marbl/CHM13 and aligned the reads with length longer than 100 kb to the T2T reference genome (chm13 v2.0) by minimap2 with parameters “-t 32 -map-ont -o”. For comparison, RAfilter was run with different numbers of threads such as 8, 16, 32 and 48. The program was run on a server with two EPYC 7H12 CPUs (each with 64 cores and 2.6 GHz) and 1024 GB memory. The usage time and CPU percentage were used to evaluate the time efficiency and the maximum memory usage was used to evaluate the space efficiency of RAfilter.

Fig. 4 shows that RAfilter with 48 threads was more than 4 folds faster than the one with 8 threads, and the CPU percentage increased linearly as the number of threads used. With different number of threads, the maximum memory usage was almost same. All these results show the good parallelization performance of RAfilter.

**Table 1:** Performance of RAfilter on real rice data.

	**HiFi**	**ONT**
**File Size (GB)**	51	34
**#Reads**	1 554 958	204 733
**#Alignments**	2 199 373	333 641
**#Filtered Alignments**	443 461	116 738
**Filtering Rate**	65.4%	86.0%
**Running Time (h:m:s)**	8:26:29	2:36:52
**Maximum Memory (GB)**	53	44

### Whole genome evaluation

To test the performance of RAfilter on whole genome level, we generated two simulated datasets (HiFi and ONT) of the whole rice genome J4155S downloaded from the project of PRJCA008812 [15] at the National Genomics Data Center, and aligned the reads to the reference genome by minimap2. Fig. 5**A, B, C** show that the performance of RAfilter on the whole genome is different from that on the only-repeat-related datasets. Both precisions and sensitivities show very high variation with different kMAPQ thresholds. We also find that the performance on ONT dataset is better than that on HiFi dataset, which is different from the results on only-repeat-related datasets. Although not for sure, we believe the reason could be related to the proportion of repeat-related alignments. In the whole genome, the vast majority of the alignments are not in the repetitive regions, and minimap2 generated much fewer false-positive alignments on HiFi data than ONT data due to the difference in sequencing error rate. It could be easier for RAfilter to detect from a much larger set of false-positive alignments (ONT) than a smaller set (HiFi).

To test the performance of RAfilter on the alignments left from normal MAPQ (another quality score of alignment) based filtering, we first filtered them by the MAPQ thresholds 10, 20, 30, 40 and 50 respectively, and then do the further filtering by RAfilter with kMAPQ thresholds 10 and 12 for ONT and HiFi alignments respectively. The results from Fig. 5**C, D** show that only a small proportion of the false-positive alignments can be removed by MAPQ based filtering, and increasing the threshold from 10 to 50 did not change much. However, RAfilter was able to remove about half (or more) more false-positive alignments.

### Performance on real rice data

Finally, we tested the performance of RAfilter on a real rice genome J4155S. The real HiFi and ONT reads and the corresponding reference genome (contigs) were downloaded from CRR453541 and CRR453543 under the project [15] (PRJCA008812). The results are shown in Table 1. After aligning the 1 554 958 HiFi reads and 304 733. ONT reads to the reference genome by minimap2, 2 199 373 and 333 641 alignments were obtained. From the outputs of minimap2, RAfilter filtered 443 461 (HiFi) and 116 738 (ONT) false-positive alignments respectively with acceptable time and memory usages. Although there is no “ground truth” for real dataset to evaluate the accuracy, it is obvious to see that the numbers of detected false-positive alignments are close to the differences between number of alignments and number of reads for both HiFi and ONT data, which proves the correctness of RAfilter to some extent.

## Discussion

We presented RAfilter, a new algorithm for filtering false-positive alignments in repetitive genomic regions. We found that RAfilter is able to detect false-positives in both tandem repeats and interspersed repeats with high or acceptable precisions and sensitivities and high time and space efficiencies. Nevertheless, there are a number of areas that our methods and approaches can be further improved. First, due to the high sequencing error rate, a large proportion of rare *k-*mers disappear in ONT reads leading to the lack of usable *k-*mers in some repetitive regions. Thus, the current version of RAfilter is not able to work well on these regions. One possible solution is to take advantage of the rare combinations of non-rare *k-*mers. More specifically, a motif that contains two or more non-rare *k-*mers may appear in the whole genome for only one or small number of times, and can be used as a marker in repetitive regions which is lack of rare *k-*mers.

Second, when calculating kMAPQ score, current version of RAfilter only considers the *k-*mer sequences and *k-*mer orders. More information such as *k-*mer orientations and *k-*mer distances can be used in the calculation to improve the accuracy in the future.

Last but not least, although a series of techniques are used to improve the time efficiency and space efficiency in RAfilter, the time and space requirements of large genomes are still not acceptable on personal computers due to the huge number of rare *k-*mers. One possible solution is to use a selected set of rare *k-*mers instead of the whole set. In some regions that contain far-more-than-enough rare *k-*mers, a sub-sampling strategy can be tried to reduce the number and thus save the time and space.

## Methods

RAfilter is composed of two phases: rare *k-*mer identification and positioning, and alignment filtering. In the first phase, the rare *k-*mers are identified and positioned on all DNA sequences related. In the second phase, the *k-*mer position information is searched for each alignment and used to decide if the alignment is false-positive. The input of RAfilter contains a set of alignments and the related sets of reference sequences and query sequences, and the output is the same set of alignments with kMAPQ scores assigned. The pipeline of RAfilter algorithm is illustrated in Fig. 1.

### Rare *k-*mer identification and positioning

In the first phase, we identify the rare *k-*mers and position them on the input reference and query sequences. First of all, we define rare *k-*mer formally as the *k-*mer which appears in the whole genome for less than or equal to *m* (e.g. 3) times. Due to the lack of whole genome sequence, we use the whole set of reference sequences to replace whole genome, because, in most situations of genome assembly, the reference sequences are non-redundant ones such as contigs and scaffolds which contain almost the same set of *k-*mers with same frequencies as the real genome. To identify rare *k-*mers, Jellyfish [12] v2.3.0 (https://github.com/gmarcais/Jellyfish) is used to collect all *k-*mers in reference sequences and count their appearing and then the ones with counts less than or equal to *m* are kept as rare *k-*mers.

Then we search in the reference and query sequences to find the positions that the identified rare *k-*mers appear at. To speed up this process and save space, RAfilter uses bit operations. First, the *k-*mers are converted into Unit64 integers by changing A, T, C, G to 2-bits 00, 11, 01, 10 respectively (with which the 2-bit of each nucleotide can be fast calculated through a bitwise NOT operation on the 2-bit of its complementary nucleotide), and then the integers are stored in a hash table. Second, for each reference or query sequence, a sliding window of length *k* (the *k* of *k-*mer) moves from the 5′ end to 3′ end. At each position, the *k-*mer in the window is searched in the hash table of rare *k-*mers by its corresponding Unit64 integer to see if any rare *k-*mer appears at this position. With bit operation, when moving the sliding window from one position to next, RAfilter generates the Unit64 integer of the new *k-*mer by taking advantage of that of the last *k-*mer, instead of the time-consuming recalculation. More specifically, to generate the new integer, we just need to left-shift the binary form of the old integer by 2 bits, add the 2 bits corresponding to the new nucleotide to the end, and mask the first 64–2 *k* bits. At each position, besides the *k-*mer in the window, we also check its reverse complement *k-*mer. Similarly, the Unit64 integer of reverse complement *k-*mer can be calculated by right-shifting the binary form with adding and masking. To speed up, this whole process was implemented with multi-threading technology. Since the tasks of different sequences are independent, RAfilter assigns each of them to one thread.

### Fast *k-*mer information searching

After positioning, RAfilter gets a rare *k-*mer list for each reference or query sequence with positions, sequences and orientations annotated. To decide if one alignment is false-positive or not, we need to obtain the corresponding rare *k-*mer sub-list for each of the two aligned sub-sequences from the rare *k-*mer position database built, which is not trivial due to the huge amount of rare *k-*mers and sequences in the whole genome. To efficiently search for a sub-list of rare *k-*mers with the given sequence ID, aligning starting position and aligning ending position, RAfilter uses a series of techniques to speed up and reduce the memory consumption. First of all, a file index is built for fast obtaining the rare *k-*mer information about the given sequence. The list of *k-*mers of each sequence is saved in the database file as a line, and the address of each line starting position is calculated by counting the total number of bytes in the previous lines. A hash table (file index) is used to save the mapping from sequence ID to the corresponding line starting address, so that RAfilter is able to find this line (the whole list of rare *k-*mers of the given sequence) by sequence ID within O [1] time. Next, we consider obtaining the sub-list of rare *k-*mers corresponding to the aligned sub-sequence from the whole list with high time and space efficiency. For reference sequence, RAfilter moves the whole list of rare *k-*mers from the file to the memory, and use a binary search algorithm (O(log*n*) time complexity) to search for the aligning starting position and aligning ending position. Since the alignments are sorted by the reference ID, one movement of rare *k-*mer list to memory solves all the alignments related to the same reference sequence, which significantly reduce IO times and save the space. For query sequence, instead of obtaining the sub-list from the whole list in memory, RAfilter implements the external binary search algorithm by file operations and directly does it in the file.

### kMAPQ calculation

For each alignment, after obtaining the two sub-lists of rare *k-*mers corresponding to the two aligned sub-sequences, RAfilter takes advantage of them to calculate the kMAPQ score which evaluates the correctness of this alignment. More specifically, we need to get a value to evaluate the similarity of the two lists of *k-*mers. The most straightforward idea is to model this problem into a theoretical problem in computer science called Longest Common Subsequence (LCS) which tries to obtain a sequence from two input sequences which satisfies 1) this sequence is a sub-sequence of both of the two input sequences; and 2) the number of elements in this sequence is the largest among all the sequences satisfying 1). After obtaining the LCS of the two lists of *k-*mers, the length of LCS can be used to evaluate their similarity. However, due to the high time complexity O(*n* [2]) for obtaining LCS, the running time is not acceptable for many long alignments which contains huge numbers of rare *k-*mers. To speed up, instead of LCS, we model this practical problem into another theoretical problem called Longest Increasing Subsequence (LIS) which can be solved by the algorithm with O(*n*log*n*) time complexity. LIS problem tries to obtain a sequence from an input digital sequence which satisfies 1) this sequence is a sub-sequence of the input sequence; 2) this sequence is an increasing sequence and 3) the number of elements in this sequence is the largest among all the sequences satisfying 1) and 2). First of all, RAfilter obtains the common set of *k-*mers that appear in both of reference and query sub-lists. Second, a digital sequence of positions of the common *k-*mers in reference is generated and the order of the sequence depends on the order of common *k-*mer in query. Third, RAfilter obtains the LIS of this digital sequence by an algorithm combining greedy strategy and binary search. Finally, the kMAPQ score can be calculated by the length of LIS *l_LIS_* and the lengths of reference and query sub-lists *l*_r_ and *l*_q_ as follows.

$$ kMAPQ=-10{\mathit{\log}}_{10}\left(\frac{l_{LIS}}{\mathit{\min}\left\{{l}_r,{l}_q\right\}}\right) $$

### Performance evaluation

In this paper, we used precision and sensitivity to evaluate the accuracy of RAfilter. TP (True Positive), FP (False Positive) and FN (False Negative) are defined as follow: TP: the number of false-positive alignments correctly detected by RAfilter; FP: the number of correct alignments wrongly detected by RAfilter as false-positive alignments; FN: the number of false-positive alignments wrongly detected by RAfilter as correct alignments. With the definition of TP, FP and FN, we define the precision and sensitivity as follow.

$$ precision=\frac{TP}{TP+ FP} $$

$$ \begin{align*} sensitivity=\frac{TP}{TP+ FN} \end{align*}$$

## Acknowledgements

This work was supported by Shenzhen Science and Technology Program (Grant No. RCBS20210609103819020) and National Natural Science Foundation of China (Grant No. 32100501). The authors thank Shengcheng Zhang for the discussions about high-efficient implementation.

## Author Contributions

W.P., J.Y. and Y.Y. designed this study. J.Y., X.Z. and H.J developed and tested RAfilter. J.Y., Y.Y and W.P. wrote the paper.

## Data Availability

The software, source codes and manual of RAfilter can be freely downloaded at https://github.com/panlab-bioinfo/RAfilter.git.

## Conflict of interests statement

None declared.

## References

[1] Sohn J , Nam J-W. The present and future of de novo whole-genome assembly. *Brief Bioinform*. 2018;19:23–40. 27742661 10.1093/bib/bbw096

[2] Hon T , Mars K, Young G et al. Highly accurate long-read HiFi sequencing data for five complex genomes. *Sci Data*. 2020;7:399. 33203859 10.1038/s41597-020-00743-4 PMC7673114

[3] Cheng H , Concepcion GT, Feng X et al. Haplotype-resolved de novo assembly using phased assembly graphs with hifiasm. *Nat Methods*. 2021;18:170–5. 33526886 10.1038/s41592-020-01056-5 PMC7961889

[4] Kolmogorov M , Yuan J, Lin Y et al. Assembly of long, error-prone reads using repeat graphs. *Nat Biotechnol*. 2019;37:540–6. 30936562 10.1038/s41587-019-0072-8

[5] Nurk S , Walenz BP, Rhie A et al. HiCanu: accurate assembly of segmental duplications, satellites, and allelic variants from high-fidelity long reads. *Genome Res*. 2020;30:1291–305. 32801147 10.1101/gr.263566.120 PMC7545148

[6] Ruan J , Li H. Fast and accurate long-read assembly with wtdbg2. *Nat Methods*. 2020;17:155–8. 31819265 10.1038/s41592-019-0669-3 PMC7004874

[7] Nurk S , Koren S, Rhie A et al. The complete sequence of a human genome. *Science*. 2022;376:44–53. 35357919 10.1126/science.abj6987 PMC9186530

[8] Deng Y , Liu S, Zhang Y et al. A telomere-to-telomere gap-free reference genome of watermelon and its mutation library provide important resources for gene discovery and breeding. *Mol Plant*. 2022;15:1268–84. 35746868 10.1016/j.molp.2022.06.010

[9] Mascher M , Wicker T, Jenkins J et al. Long-read sequence assembly: a technical evaluation in barley. *Plant Cell*. 2021;33:1888–906. 33710295 10.1093/plcell/koab077 PMC8290290

[10] Hou X , Wang D, Cheng Z et al. A near-complete assembly of an Arabidopsis thaliana genome. *Mol Plant*. 2022;15:1247–50. 35655433 10.1016/j.molp.2022.05.014

[11] Li H . New strategies to improve minimap2 alignment accuracy. *Bioinformatics*. 2021;37:4572–4. 34623391 10.1093/bioinformatics/btab705 PMC8652018

[12] Marcais G , Kingsford C. Jellyfish: a fast *k-*mer counter. *Tutorialis E Manuais*. 2012;1:1–8.

[13] Ono Y , Asai K, Hamada M. PBSIM: PacBio reads simulator—toward accurate genome assembly. *Bioinformatics*. 2013;29:119–21. 23129296 10.1093/bioinformatics/bts649

[14] Ono Y , Asai K, Hamada M. PBSIM2: a simulator for long-read sequencers with a novel generative model of quality scores. *Bioinformatics*. 2021;37:589–95. 32976553 10.1093/bioinformatics/btaa835 PMC8097687

[15] Zhang Y , Fu J, Wang K et al. The telomere-to-telomere gap-free genome of four rice parents reveals SV and PAV patterns in hybrid rice breeding. *Plant Biotechnol J*. 2022;20:1642–4. 35748695 10.1111/pbi.13880 PMC9398309

